# New Insights Into the Role of Placental Aquaporins and the Pathogenesis of Preeclampsia

**DOI:** 10.3389/fphys.2018.01507

**Published:** 2018-10-30

**Authors:** Natalia Szpilbarg, Nora A. Martínez, Mauricio Di Paola, Julieta Reppetti, Yollyseth Medina, Abril Seyahian, Mauricio Castro Parodi, Alicia E. Damiano

**Affiliations:** ^1^Laboratorio de Biología de la Reproducción, Instituto de Fisiología y Biofísica Bernardo Houssay (IFIBIO)-UBA-CONICET, Facultad de Medicina, Universidad de Buenos Aires, Buenos Aires, Argentina; ^2^Cátedra de Biología Celular y Molecular, Departamento de Ciencias Biológicas, Facultad de Farmacia y Bioquímica, Universidad de Buenos Aires, Buenos Aires, Argentina

**Keywords:** extravillous trophoblast, villous trophoblast, AQP3, AQP9, human placenta, preeclampsia

## Abstract

Accumulated evidence suggests that an abnormal placentation and an altered expression of a variety of trophoblast transporters are associated to preeclampsia. In this regard, an abnormal expression of AQP3 and AQP9 was reported in these placentas. Recent data suggests that placental AQPs are not only water channel proteins and that may participate in relevant processes required for a normal placental development, such as cell migration and apoptosis. Recently we reported that a normal expression of AQP3 is required for the migration of extravillous trophoblast (EVT) cells. Thus, alterations in this protein might lead to an insufficient transformation of the maternal spiral arteries resulting in fluctuations of oxygen tension, a potent stimulus for oxidative damage and trophoblast apoptosis. In this context, the increase of oxygen and nitrogen reactive species could nitrate AQP9, producing the accumulation of a non-functional protein affecting the survival of the villous trophoblast (VT). This may trigger the exacerbated release of apoptotic VT fragments into maternal circulation producing the systemic endothelial dysfunction underlying the maternal syndrome. Therefore, our hypothesis is that the alteration in the expression of placental AQPs observed at the end of gestation may take place during the trophoblast stem cell differentiation, disturbing both EVT and VT cells development, or during the VT differentiation and turnover. In both situations, VT is affected and at last the maternal vascular system is activated leading to the clinical manifestations of preeclampsia.

## Introduction

Preeclampsia is a pregnancy complication characterized by high blood pressure and proteinuria which usually begins abruptly after 20 weeks of gestation. This condition is exclusively for human gestation and affects 7–10% of pregnancies worldwide (Giachini et al., 2017). In Latin-American countries, it is estimated that 26% of maternal deaths are related to preeclampsia (Khan et al., 2006; Abalos et al., 2013).

The consequences of preeclampsia are not limited to the pregnancy and may conduce to maternal permanent vascular and metabolic damage and future heart diseases. Moreover, emerging evidence suggests that the adaptation to an adverse intrauterine environment may also affect the adult life of the newborn (Giachini et al., 2017).

Despite the importance of preeclampsia and several decades of extensive research, its etiopathogenesis remains unclear. It is well accepted that defects in placentation are the main predisposing factors for preeclampsia (Myatt, 2002; Huppertz, 2008; Hawfield and Freedman, 2009). However, the molecular basis of the abnormal placental development has not been sufficiently clarified yet. Several theories have been proposed to elucidate the origins of preeclampsia but none of them can explain the variability of cases. In fact, preeclampsia is a very heterogeneous syndrome and is classified by the severity of the disease as mild, moderate, and severe and by the time of delivery as early and late onset preeclampsia, with or without fetal growth restriction (Huppertz, 2008).

The most cited hypothesis on the etiology of preeclampsia considers this syndrome as a two-stage disorder (Hawfield and Freedman, 2009). The first stage is characterized by an insufficient transformation of the maternal spiral arteries resulting in a decrease in blood supply to the fetoplacental unit. Consequently, fluctuation in oxygen levels may increase the damage of the villous trophoblast (VT) concluding in the maternal endothelial dysfunction and the clinical manifestations of preeclampsia. Nevertheless, the link between preeclampsia and a failure of trophoblast invasion has recently raised serious doubts because the abnormal transformation of the uterine spiral arteries gives rise to the maternal symptoms only in some women (Huppertz, 2008, 2018). That is why, the current view regarding the etiology of preeclampsia has been recently updated toward the dysregulation of VT turnover.

In this regard, it was proposed that failures in the differentiation of the villous syncytiotrophoblast could accelerate the shedding of apoptotic syncytial aggregates (Norah et al., 2013). The detachment from the apical syncytiotrophoblast membrane of these structures into the maternal circulation, induce an antiangiogenic environment and enhance the maternal systemic inflammatory response producing the endothelial dysfunction. (Huppertz, 2008, 2018; Norah et al., 2013). Even more, a failure in the undifferentiated trophoblast stem cells, may affect VT and EVT lineage resulting also in an inadequate transformation of the uterine spiral arteries. In this condition, preeclampsia is associated with growth restriction (Huppertz, 2008, 2018).

Villous syncytiotrophoblast is involved in the fetal-maternal oxygen and nutrient exchange, and many placental transporters and channels are altered in preeclamptic placentas (Damiano et al., 2006; del Monaco et al., 2006; Castro-Parodi et al., 2009; Dietrich et al., 2013; Martínez et al., 2016; Szpilbarg and Damiano, 2017).

## Water Transport and Placental Aquaporins

Fetal water requirements increase throughout gestation accompanying fetal growth. Emerging evidence show that water can pass across the placenta by transcellular and paracellular pathways, but the molecular mechanisms of these processes are not clarified yet. Previous research using isolated membrane vesicles showed that water moves through the syncytiotrophoblasts by lipid diffusion (Edwards et al., 1993; Jansson and Illsley, 1993; Jansson et al., 1999) discarding the transcellular route and the participation of water-channel cell membrane proteins known as aquaporins (AQPs).

However, in 2001 we described that AQP3 and AQP9 are present in the apical membrane of the human syncytiotrophoblast (Damiano et al., 2001). Both proteins are members of the aquaglyceroporin subfamily and allow the transport of water, urea, and glycerol. Exceptionally, AQP9 also facilitates the flux of neutral solutes such as monocarboxylates, purines, and pyrimidines (Ishibashi et al., 1998; Tsukaguchi et al., 1998). Uptake experiments showed that these AQPs may mediate the transcellular water, urea, and glycerol transport in human placenta (Damiano et al., 2006; Table 1).

**Table 1:** Expression and functionality of AQP3 and AQP9 in normotensive and preeclamptic placentas.

	Normotensive Placentas	Preeclamptic Placentas
AQP3 EXPRESSION	Yes (Damiano et al., 2001; Zhu et al., 2009)	Yes, decreased (Szpilbarg and Damiano, 2017)
AQP3 LOCALIZATION	Apical membrane of syncytiotrophoblast cells (Damiano et al., 2001; Zhu et al., 2010)	Apical membrane of syncytiotrophoblast cells (Szpilbarg and Damiano, 2017)
AQP9 expression	Yes (Damiano et al., 2001)	Yes, increased (Damiano et al., 2006)
AQP9 localization	Apical membrane of syncytiotrophoblast cells (Damiano et al., 2001; Zhu et al., 2010)	Apical and basal membranes, and cytoplasm of syncytiotrophoblast cells (Damiano et al., 2006)
WATER UPTAKE, *pmol. g^-1^.min^-1^*		
Control	76 ± 6	47 ± 10^∗^
+ HgCl_2_ 0.3mM	48 ± 7^#^	36 ± 12
	(Damiano et al., 2006)	(Damiano et al., 2006)
Mannitol Uptake, *pmol. g^-1^.min^-1^*		
Control	14.6 ± 0.3	5.5 ± 0.6^∗^
+ HgCl_2_ 0.3mM	7.8 ± 0.8^#^	6.0 ± 2.3
	(Damiano et al., 2006)	(Damiano et al., 2006)


*^∗^*P* < 0.05 normotensive vs. preeclamptic placentas. ^#^*P* < 0.05 in the absence vs. in the presence of HgCl_2_, a general blocker of AQPs.*

However, in preeclamptic placentas we found that the expression of AQP9 increased and the cellular distribution of this protein changed, being localized not only in the apical and basal membranes but also in the cytosol of the syncytiotrophoblasts (Damiano et al., 2006). In contrast, we have recently reported that AQP3 expression considerably decreased in preeclamptic placentas compared to normal ones (Szpilbarg and Damiano, 2017).

Regarding AQPs functionality, water, and mannitol uptakes also decreased in preeclamptic placentas, compared to normal ones and were not sensitive to HgCl_2_ (Damiano et al., 2006; Table 1).

Although the reduced uptake of water may be associated to AQP3 expression, the lack of sensitivity to HgCl_2_ and the reduced uptake of mannitol, which can only permeate through AQP9, may suggest that both proteins are not functional in preeclamptic placentas.

Given that there is no evidence that the water fetal-maternal flux is altered in preeclampsia, the classical role of these placental AQPs exclusively in facilitation of trans-epithelial fluid transport has been called into question (Damiano, 2011; Martínez and Damiano, 2017). Recently, unexpected cellular roles of AQPs were reported, including organelle physiology, proliferation, apoptosis, and cell migration (Verkman, 2011; Kitchen et al., 2015). All of them are related to temporary cell volume changes.

It is important to note that human placenta is hemomonochorial, in which the syncytiotrophoblast, a single layer of polarized epithelium separates the maternal and fetal circulation (Stulc, 1997). Because of the similarities in the fetal-maternal transfer barrier, the guinea pig is the only animal model established to study placental transport. Despite of this, several studies were carried out in mice, rats, and sheep reporting changes in the expression of AQPs during pregnancy that could be associated with changes in placental water transfer and amniotic fluid homeostasis (Liu et al., 2004; Beall et al., 2007; Belkacemi et al., 2011). In addition, AQP3-knockout mice were viable and developed normally, but no studies ruled out placental or pregnancies alterations in these mice (Verkman, 2006; Lei et al., 2017).

On the other hand, preeclampsia is a disorder unique to human pregnancy, and no animal model could reproduce the complete syndrome. Taken into account these difficulties, the role of AQPs in human preeclamptic placenta was studied by *in vitro* models (Martínez and Damiano, 2017).

## Oxygen Regulation of Placental Aquaporins

It is well known that trophoblast differentiation is regulated by oxygen (Genbacev et al., 1997; James et al., 2006). During normal pregnancies, placentation occurs in a relatively hypoxic environment. Hypoxia inducible factor-1α (HIF-1α) may contribute to the adaptation of the placenta to fluctuations in the oxygen tensions (Patel et al., 2010).

In pathological conditions, intermittent hypoxia because of the decreased maternal blood flow into the intervillous space may produce an ischemia/reperfusion insult in the placenta (Hung and Burton, 2006).

Since hypoxia controls the expression of many genes involved in cell adaptation to stress, the regulation of placental AQPs by oxygen was studied. In normal placental explants exposed to oxygen deprivation, HIF-1α was detected and AQP9 protein drastically decreased (Castro-Parodi et al., 2013). However, in explants cultured in hypoxia/reoxygenation, HIF-1α was undetectable and AQP9 showed a significant increased. In this condition, we also observed that AQP9 was localized in the apical and basal membranes and in the cytosol of the syncytiotrophoblast as previously reported in preeclamptic placentas (Damiano et al., 2006; Castro-Parodi et al., 2013).

Regarding AQP3, hypoxia decreased the protein expression which was abnormally localized in the cytosol. After reoxygenation, AQP3 returned to the apical membrane of the syncytiotrophoblast, but its expression was not restored to control levels (Szpilbarg et al., 2016).

## Placental Aquaporins and Apoptosis

Trophoblast apoptosis increases progressively during gestation. This physiological process is required for normal turnover of VT and comprises the fusion of the mononuclear cytotrophoblast cells into the multinucleate syncytium (Smith and Baker, 1999; Huppertz et al., 2006; Sharp et al., 2010). These events lead to the release into maternal circulation of syncytial aggregates, which progressively increase during normal pregnancy and do not harm the mother (Burton and Jones, 2009).

In pregnancies complicated by preeclampsia, VT apoptosis is exacerbated compared to normotensive pregnancies (Sharp et al., 2010). It was proposed that an altered balance between proliferation and apoptosis could increase the formation of syncytial aggregates shedding into the maternal circulation. This favors the immunological and inflammatory processes of the mother and promotes the systemic endothelial dysfunction (Huppertz, 2008; Norah et al., 2013; Roland et al., 2016).

Concerning the role of AQPs in the programmed cell death, Jablonski et al. (2004) demonstrated that AQPs may mediate the loss water and subsequent cell shrinkage in the apoptotic cells. The proposed mechanism is that the K^+^ efflux and the intracellular K^+^ depletion generate an osmotic gradient that drives water out of the cell, a process known as apoptotic volume decrease (AVD). Inactivation of AQPs after AVD and the continuous efflux of ions K^+^ decrease the ionic strength of the cytoplasm and activate the apoptotic caspases. (Chen et al., 2008).

Given this background, we studied the role of AQPs in the VT apoptosis. We observed that only the inhibition of AQP3 abrogates the apoptotic response of these cells (Szpilbarg et al., 2016). Thus, we provided evidence that AQP3 may be important in the regulation of VT apoptosis and consequently an abnormal expression of this protein could alter this tightly regulated process.

Along with this idea, we expected that an altered AQP3 may be one of the key factors in the development of preeclampsia. In this context, we assumed that the increase in trophoblast apoptosis observed in preeclampsia might correlate with an increase in AQP3, but we found a reduced expression of this protein in these placentas (Szpilbarg and Damiano, 2017). Consequently, the role of AQP3 in the apoptosis of the VT in preeclampsia remains unclear.

One possibility is that AQP3 decreases as an adaptive response of the trophoblast to reduce the apoptotic events observed in preeclampsia.

Another possible explanation is that the damage to the syncytiotrophoblast membranes produced by the intermittent hypoxia may create an unfavorable environment for AQP3 insertion into the plasma membrane, increasing its degradation. In fact, the apical membranes of syncytiotrophoblast from preeclamptic placentas are more rigid than normal ones, due to an increase in sphingomyelin that reduces the number of caveolae and the expression of Caveolin-1 (Levi et al., 2016). Moreover, analysis of the primary structure of AQPs revealed a putative caveolin-1-binding site which is required for AQPs functionality (Jablonski and Hughes, 2006). Consequently, an altered lipid composition may disrupt the ability of sphingomyelin and cholesterol to assemble into caveolae in the apical leaflet of the bilayer, affecting protein expression and cell signaling (Levi et al., 2016).

Regarding AQP9, we found that its blocking did not prevent the apoptotic response of the trophoblast (Szpilbarg et al., 2016), discarding a direct role of the AQP9 in this process. Furthermore, despite the increase in AQP9 protein expression, we observed a lack of its functionality for water and monocarboxylates in preeclamptic placentas (Damiano et al., 2006).

The placenta is a main source of reactive oxygen (ROS) and nitrogen (RNS) species which can nitrate tyrosine sites of proteins by enhancing the production of peroxynitrite (Webster et al., 2008; Myatt, 2010). Nitration of proteins can cause a loss of function. In recent studies (unpublished), we observed an increase of 3-nitrotyrosine AQP9 in preeclamptic placentas. In this context, we propose that this nitrated protein may impair the transfer of lactate, an end-product of anaerobic glycolysis.

Evidence suggests that AQP9 could play a role both in energy metabolism and in the clearance of free radicals. Recently, it was reported in preeclamptic placentas, a significant decrease in the protein expression and the function of GLUT-1 (Lüscher et al., 2017). Therefore, faced with the reduction of the glucose passage, the trophoblast might be forced to use another source of energy like lactate (Lüscher et al., 2017).

Lactate is an energy substrate and can also be involved in scavenging ROS as a source of NADH (Schurr and Gozal, 2011). Although the expression of monocarboxylate symporters MCT1 and MCT4 has been found in syncytiotrophoblast membranes (Settle et al., 2004), no data was found about their expression in preeclamptic placentas. For that reason, we hypothesize that the lack of functionality of AQP9 may increase ROS accumulation and adversely affect the survival of the trophoblast cells, like in other tissues (Miki et al., 2013; Akashi et al., 2015). Therefore, AQP9 may contribute to exacerbate the trophoblast apoptosis in an indirect manner.

In addition, supraphysiological concentrations of peroxynitrite can induce a decrease of protein degradation rates by the proteasome (Grune et al., 1998). Subsequently, we surmise that the changes in the cellular distribution of AQP9, observed in preeclamptic placentas (Damiano et al., 2006), may be due to the increase of this non-functional nitrated protein that cannot be degraded in the proteasome and accumulates in the cytosol of the syncytiotrophoblasts.

Given that preeclampsia is a syndrome of early placentation, we cannot discard that the abnormal expression of AQP3 and AQP9 observed at the end of gestation may be a consequence of a failure in the differentiation of the trophoblast stem cell. In this regard, we investigated the role of these proteins at early stages of placenta development.

## Placental Aquaporins and Migration

To date, many AQPs were described in the blastocyst and in the early and term placenta (Escobar et al., 2012; Xiong et al., 2013). AQP3, AQP1, and AQP9 are the most abundant AQPs expressed in chorionic villi from first trimester suggesting that they may have a key role in the normal fetal growth and homeostasis.

A highly synchronized trophoblast differentiation, proliferation, and invasion are necessary to achieve a successful pregnancy. Nowadays, the molecular mechanisms that lead these complex processes remain unknown. Trophoblast cells differentiate to invasive extravillous trophoblast (EVT) or fuse to form the syncytium (Velicky et al., 2016). Thereby, EVT cells change their epithelial phenotype to an invasive mesenchymal phenotype. These events resemble the general epithelial-mesenchymal transition process and allow EVT to invade the endometrium. (DaSilva-Arnolda et al., 2015; Davies et al., 2016). In these processes, mechanisms of migration and invasion displayed by trophoblast and malignant cells are similar. However, unlike tumors, the trophoblast behavior is tightly controlled (Soundararajan and Jagannadha Rao, 2004; Piechowski, 2016).

Moreover, increasing evidence demonstrated that AQPs may be involved in tumor cell migration. (Hu and Verkman, 2006; Nico and Ribatti, 2011; Cao et al., 2013; Ribatti et al., 2014; Marlar et al., 2017).

Considering that preeclampsia is related to an abnormal placentation and an altered placental expression of AQP3 and AQP9 was found after the onset of the maternal syndrome, we assumed that these proteins may participate in the early stages of placental development.

Along with this idea, we explored the contribution of AQPs to the EVT cell migration.

Our findings strongly show that only the blocking of AQP3 or the silencing of its expression reduce trophoblast cells migration. AQP9 is not involved in this process (Reca et al., 2018).

We believe that in preeclampsia complicated by IUGR, the aberrant expression of AQP3 may be present already in the undifferentiated trophoblast stem cell affecting both VT and EVT pathways. However, up to now, it is not possible to determine the expression or functionality of AQPs in early placentas that late in gestation will develop preeclampsia.

## Putative Participation of AQP3 and AQP9 in the Physiopathology of Preeclampsia

Emerging data suggests that placental AQPs may participate in relevant processes required for a normal placental development, such as cell migration and apoptosis (Szpilbarg et al., 2016; Reca et al., 2018).

The alteration in the expression of placental AQPs observed at the end of gestation may take place at two different steps during the trophoblast differentiation: (i) during the trophoblast stem cell differentiation disturbing both EVT and VT cells development or (ii) during the VT differentiation and turnover.

In both situations, the expression of AQPs in VT is affected and ultimately this may contribute with the development of the clinical symptoms of preeclampsia.

**FIGURE 1:**
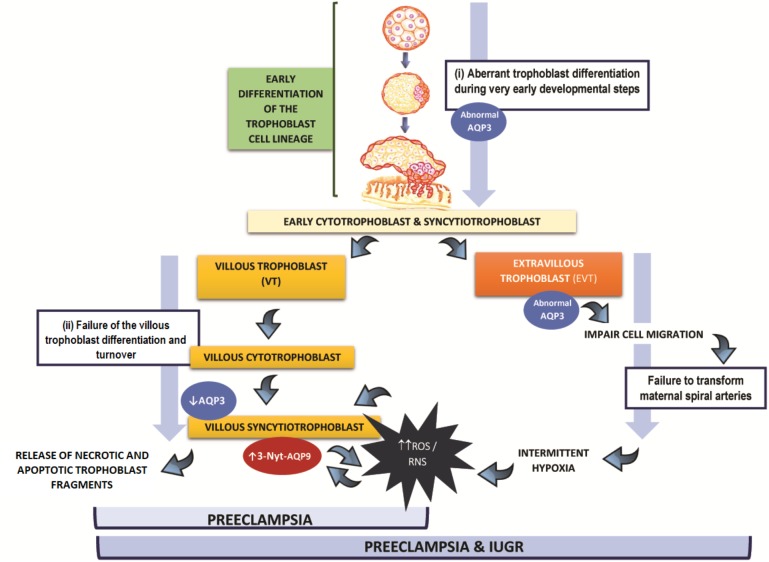
Hypothesis of the participation of AQP3 and AQP9 in the physiopathology of preeclampsia: (i) Alterations in AQPs expression may occur during the trophoblast stem cell differentiation disturbing both extravillous and villous trophoblast cells development resulting in preeclampsia with fetal growth restriction. (ii) Alterations in AQPs expression occur during villous trophoblast differentiation resulting in preeclampsia without fetal growth restriction. In both scenarios, villous trophoblast cells are affected.

Figure 1 is a theoretical representation of our hypothesis of the putative contribution of AQPs to the multiple alterations that lead to the development of preeclampsia. In the first case (i), if the aberrant expression of AQP3 occurs during the first differentiation step of the trophoblast cell lineage or slightly afterward, it may affect EVT as well as VT development. Since AQP3 is required for the appropriated migration of EVT cells (Reca et al., 2018), the abnormal expression of this protein, might lead to a shallow trophoblast invasion and insufficient transformation of the maternal spiral arteries. In this situation, intermittent hypoxia affects placentation increasing ROS and RNS, a potent stimulus for VT apoptosis (Myatt and Cui, 2004; Hung and Burton, 2006). Our hypothesis is that the increase of ROS and RNS may lead to the nitration of AQP9, resulting in a non-functional protein. In this scenario, the lack of functionality of AQP9, may impair the transfer of lactate and promote more accumulation of ROS and enhance the VT cell death. This may trigger the exacerbated release of apoptotic syncytial aggregates into maternal circulation producing the systemic endothelial dysfunction underlying the maternal syndrome. The consequences of this set of alterations may lead to preeclampsia associated to growth restriction.

On the other hand (case ii), we propose that if the abnormal expression of AQP3 takes place only in the villous pathway, it may contribute, together with AQP9, to exacerbate the oxidative damage and apoptotic response of the trophoblast, increasing the shedding of trophoblast material into maternal circulation. Finally, this may collaborate to induce the injury of endothelial cells, resulting in preeclampsia without growth restriction.

In conclusion, abnormal expression of placental AQPs may appear at different points of trophoblast differentiation affecting VT alone or VT and EVT cells. Although preeclampsia is a complex and multisystemic disorder resulting from multiple simultaneous mechanisms, AQPs may be contributing as part of this network of alterations that give rise to the diverse clinical manifestations of preeclampsia.

## Author Contributions

AD defined the research topic. AS, YM, JR, MDP, and MC prepared the draft of the manuscript. NS and AD edited the text. NS, NM, AD co-wrote the manuscript. All authors approved the last version of the manuscript.

## Conflict of Interest Statement

The authors declare that the research was conducted in the absence of any commercial or financial relationships that could be construed as a potential conflict of interest.
